# Spontaneous Renal Hemorrhage: A Case Report and Clinical Protocol

**DOI:** 10.7759/cureus.15547

**Published:** 2021-06-09

**Authors:** Olivia Antonescu, Melanie Duhamel, Brian Di Giacinto, James Spain

**Affiliations:** 1 Interventional Radiology, Aultman Hospital/Northeast Ohio Medical University, Canton, USA; 2 Interventional/Diagnostic Radiology, Aultman Hospital/Northeast Ohio Medical University, Canton, USA; 3 Radiology, Aultman Hospital/Northeast Ohio Medical University, Canton, USA

**Keywords:** interventional radiology, spontaneous renal hemorrhage, spontaneous renal hematoma, transcatheter arterial embolization

## Abstract

Spontaneous renal hemorrhage is an uncommon entity with potentially serious consequences. We present a 68-year-old female with a three-day history of progressively worsening left-sided flank pain due to spontaneous left renal hemorrhage without a history of trauma or anticoagulation. The patient’s symptoms improved with conservative management and she was discharged after several days of observation. However, the patient was readmitted the next day with progressively worsening pain due to hematoma expansion from active extravasation. On the second admission, interventional radiology successfully embolized the affected vessels and the patient improved rapidly. The hematoma decreased in size on follow-up exams but no etiology was discovered. Early arterial embolization may have improved outcomes in this case and we argue that it should be considered early in the management of all patients with spontaneous renal hemorrhage.

## Introduction

Spontaneous renal hemorrhage (SRH) or hematoma is an intraparenchymal renal hemorrhage of unknown origin in a patient without trauma or anticoagulation [1]. SRH is most commonly related to occult vascular renal tumors (angiomyolipoma or renal cell carcinoma), vasculitides (polyarteritis nodosa), or vascular malformations. A few cases are idiopathic or have been attributed to infection, uncontrolled hypertension, ruptured hemorrhagic cysts, or erosion from large renal stones [2].

Patients with SRH will classically present with Lenk’s triad of flank pain, tenderness, and “symptoms of blood loss,” including generalized fatigue, tachycardia, dizziness, and hypotension depending on the volume of blood loss. However, it can mimic many acute abdominal pathologies. As such, SRH is usually discovered incidentally on ultrasound or contrast-enhanced abdominal CT. Abdominal CTA however is the preferred imaging modality for definitive diagnosis and preprocedural planning. Laboratory findings often include anemia and hematuria.

Multiple philosophies regarding the treatment of SRH are described in the literature. Therapeutic recommendations have evolved over time and often reflect the experience and scope of practice of the authoring clinician. Initial reports by surgical subspecialties suggested radical or modified nephrectomy for all cases [2-4]. However, a more recent case series argues that a trial of conservative inpatient or even outpatient therapy may be prudent [5]. Conservative care consists of hydration and pain management with blood product replacement in patients presenting with significant anemia, and antibiotics if there is concern for an infectious etiology [6-7]. However, it should be noted that conservative treatment comes at the cost of decreased renal function compared to other courses of treatment. Selective arterial embolization is another method to control active bleeding, particularly in hemodynamically unstable patients [8]. Many authors recommend embolization as the definitive therapy given strong evidence supporting its efficacy in cases of both traumatic and non-traumatic renal hemorrhage and relatively low-risk profile [6,9-11]. However, some authors still consider embolization as a temporizing measure and second line to nephrectomy [6]. Clinical & imaging follow-up in the setting of SRH is another area of contention. In general, it is agreed that some degree of in-office and imaging follow-up is necessary until the hematoma has resolved, an etiology is elucidated, or definitive surgery is performed [5,12].

## Case presentation

**Figure 1:**
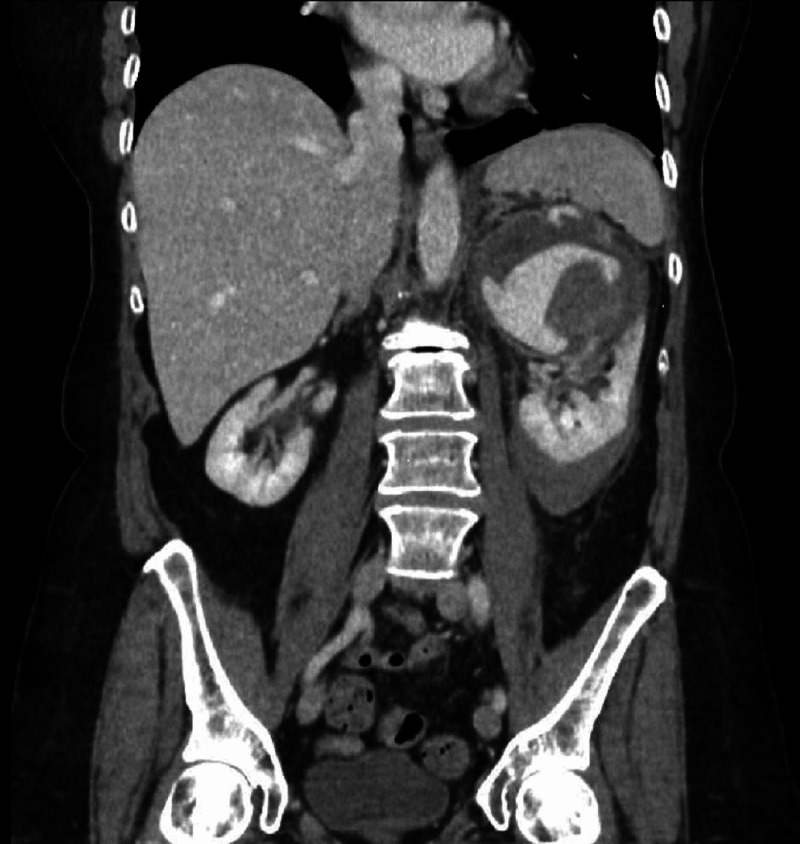
Coronal reconstruction of portal venous phase contrast-enhanced CT abdomen and pelvis demonstrates acute hemorrhage at the superior pole of the left kidney with active extravasation.

A 68-year-old woman presented to the emergency department with a three-day history of progressively worsening left-sided flank pain. There was no history of nausea, hematuria, or trauma. The patient denied a personal or family history of cancer and she denied taking any medications or supplements. Patient's vital signs were normal. Left flank tenderness was noted on physical exam but no mass was palpated. Chemistries revealed leukocytosis (12.20 x 10^9^/L white blood cells [WBC]) and anemia (11.4 g/dL hemoglobin [Hbg]). Contrast-enhanced CT abdomen and pelvis demonstrated active extravasation into a 9.2 cm hematoma at the superior pole of the left kidney (Figure 1). The patient was admitted and treated with fluid hydration and pain control. After discussing imaging with the on-call radiologist, nephrology recommended a course of conservative therapy given the clinical picture but was uncomfortable discharging the patient in the setting of active hemorrhage. A CT urogram the following day showed mild enlargement of the patient’s hematoma but failed to demonstrate an underlying tumor. The patient’s hemoglobin stabilized over the course of a four-day hospitalization and flank pain improved with opioid management. The conservative therapy trial was considered successful given the improving clinical picture. The urology service then discharged the patient on a prophylactic seven-day course of antibiotics due to concern for superinfection, and instructions to follow up with her primary care provider in one week.

**Figure 2:**
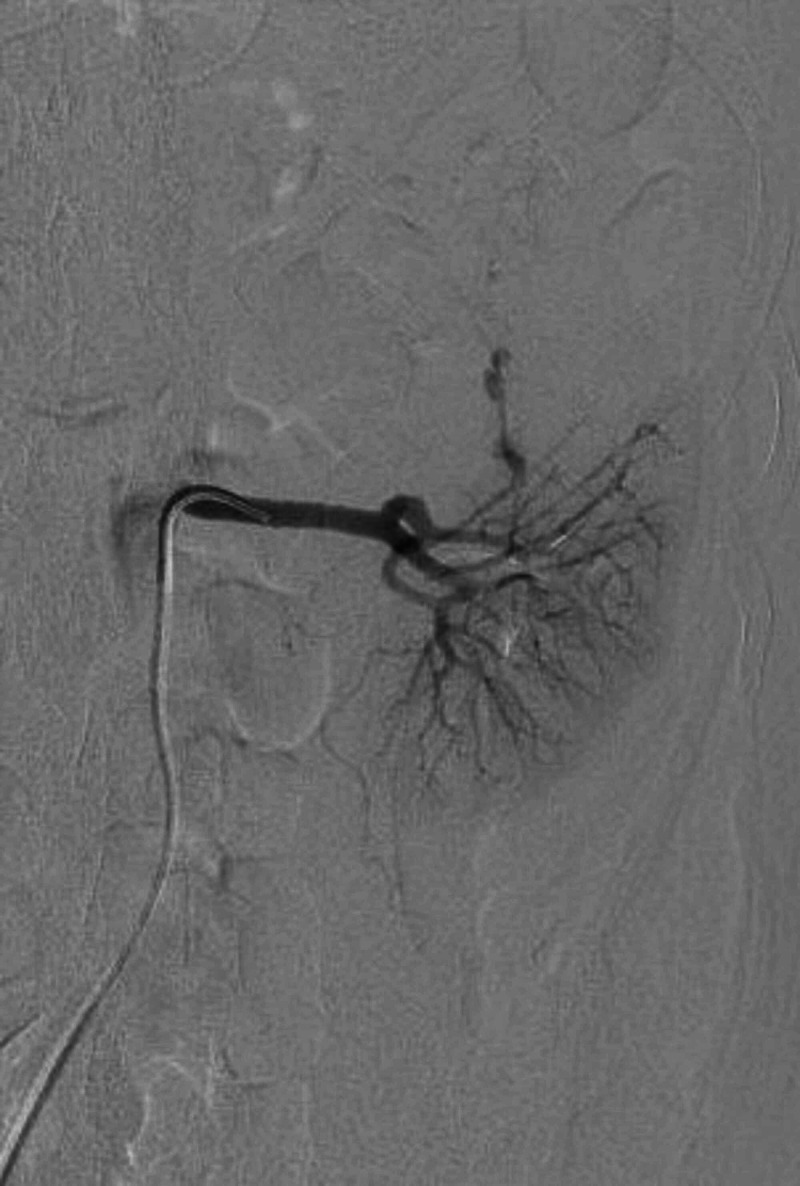
Digital subtraction angiography of the left renal artery demonstrates active extravasation from a subsegmental artery supplying the superior pole.

**Figure 3:**
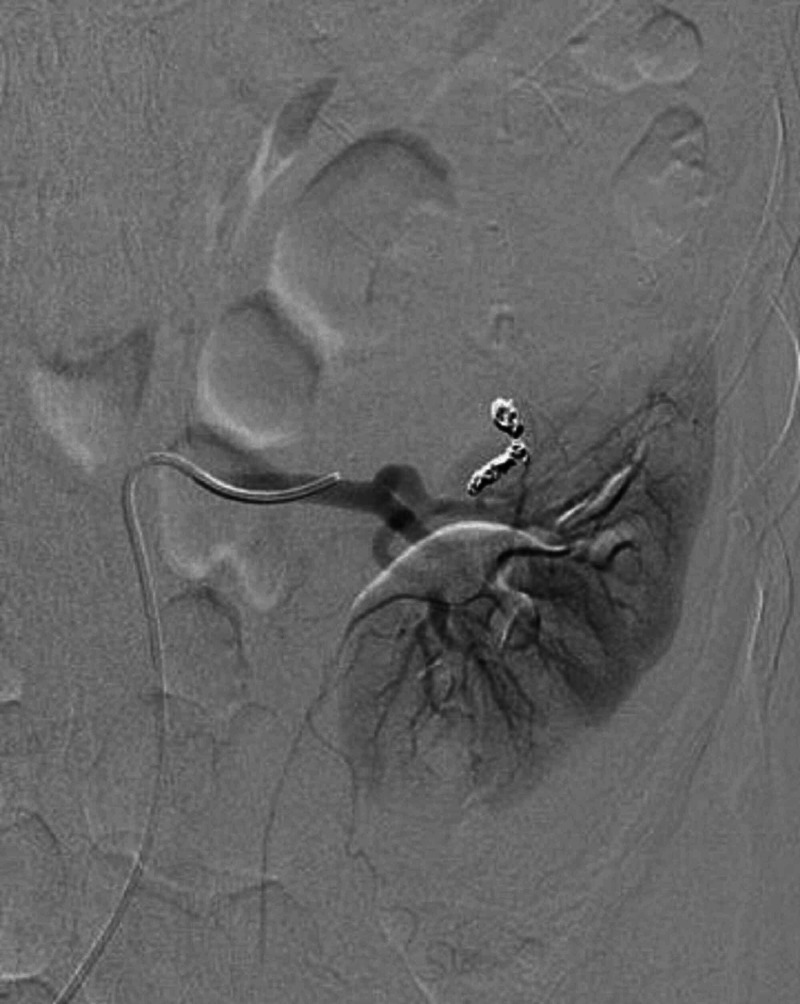
Digital subtraction angiography of the left renal artery demonstrates coil embolization of the bleeding vessel with no residual bleeding evident.

However, the patient returned to the emergency department the next day with severe intractable left flank pain. Repeat abdominal CT demonstrated progression of the renal hemorrhage with active extravasation. Interventional radiology was consulted and recommended selective renal artery embolization. Arteriogram was performed demonstrating the known hematoma and area of active bleeding (Figure 2). A 5 French C2 base catheter engaged the left renal artery and a microcatheter was advanced into the bleeding vessel, where four fibered interlocking detachable coils were placed. The post-embolization selective renal arteriograms demonstrated embolization of the bleeding vessel and preserved blood flow to the middle and lower portions of the kidney (Figure 3).

**Figure 4:**
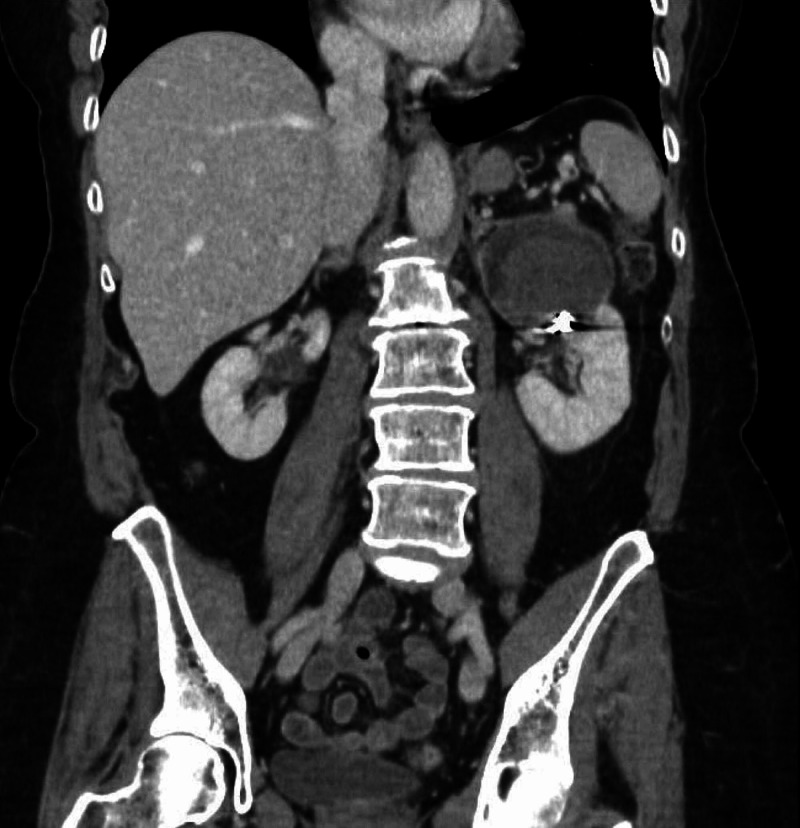
Coronal reconstruction of nephrogenic phase contrast-enhanced CT abdomen and pelvis one year following embolization demonstrate partial resorption of the left upper pole spontaneous renal hemorrhage. No renal mass is identified.

The patient reported that her pain had much improved the following day and she was discharged home with outpatient follow-up in four weeks. Follow-up contrast-enhanced computed tomography (CECT) at one month, five months, and one year demonstrated a shrinking hematoma (Figure 4). However, no mass was identified and workups for coagulopathies and angiopathies were negative.

## Discussion

SRH is a rare entity with potentially lethal consequences and broadly divergent management recommendations. Limited literature on the topic suggests that many cases may be handled conservatively with pain control and close clinical follow-up. However, more aggressive measures are often necessary given the potentially fulminant course of untreated internal hemorrhage. In our case, the patient initially presented with left flank pain, stable vital signs, and mild anemia. Based on the clinical presentation alone conservative management is appropriate. However, imaging also demonstrated active extravasation and there was no baseline hemoglobin to assess the extent of blood loss. This could be justification for more aggressive intervention early on. This conflicting logic is echoed in the literature with various authors recommending pain control and antibiotics, urgent embolization, or partial nephrectomy as initial treatment options. Conservative management must be considered when appropriate to reduce patient risk from unnecessary procedures and reduce healthcare costs. However, this must be balanced with our obligation to prevent patient suffering and provide definitive management. 

We propose a lower threshold for urgent embolization when there is clinical or imaging evidence of active hemorrhage. As illustrated in this case, patients with active hemorrhage on presentation appear more likely to demonstrate continued or recurrent bleeding resulting in progressive pain from distention of the renal capsule or surrounding fascia, hypertension related to Page kidney, or the sequelae of hemorrhagic shock. Selective renal artery embolization is a minimally invasive procedure, as compared to nephrectomy, with an extremely low risk to the patient and results in significantly improved renal function relative to conservative therapy [8]. Therefore, it seems the logical choice for initial management in this circumstance. Furthermore, delayed treatment may increase the duration of hospital stay, prolonging exposure to hospital-born infections, and possibly increase financial strain on the patient and healthcare system as a whole. This is especially true if readmission is required. The average three-day hospital admission in the United States costs approximately $30,000 adjusted for inflation in 2021 [13]. While arterial embolization procedures are usually less costly when compared to hospital readmission or equivalent open and laparoscopic procedures [14]. In this case, continued bleeding and delayed embolization resulted in prolonged patient suffering, two separate admissions, and close to a week in the hospital.

**Figure 5:**
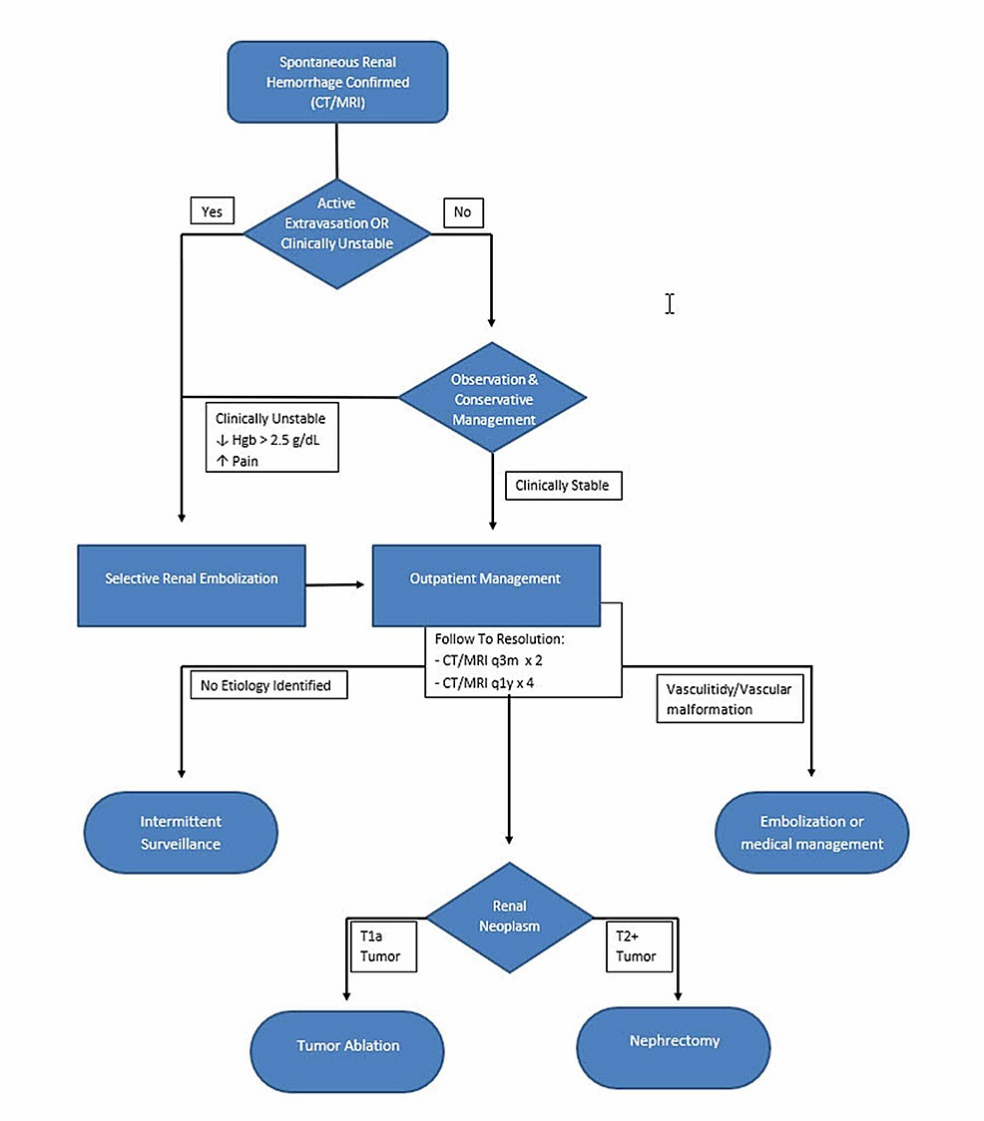
Suggested algorithm for evaluation of spontaneous renal hemorrhage. Hgb = Hemoglobin; CT = computed tomography; MRI = magnetic resonance imaging

Given the broad range of management recommendations and complexity of individual cases establishing the best course of action can be challenging. We believe that a clinical algorithm based on current literature and past experience may help establish a dialogue to optimize patient management and build a testable framework to be evaluated by future research (Figure 5).

Some authors suggest that the initial treatment of SRH should be based purely on clinical symptoms. While patient vital signs are paramount, the diagnosis of SRH cannot be made in the absence of imaging and the presence of active extravasation on CT should also guide initial management. If the patient presents with unstable vital signs or active extravasation on imaging then angiogram with embolization is indicated after appropriate stabilization [9-10]. Overnight observation with conservative management, or even outpatient management with close clinical follow-up, may be appropriate if vital signs are stable and no active extravasation is observed on imaging. If continued hemorrhage is evident clinically (progressive pain or worsening vital signs), by serial hemoglobin/hematocrit labs, or on follow-up imaging (hematoma size increase or active extravasation) then selective arterial embolization should be performed. 

Outpatient follow-up is also critical if the underlying cause cannot be identified on the initial workup. The surveillance schedule in our suggested algorithm is therefore based on National Comprehensive Cancer Network (NCCN) guidelines for surveillance of a patient with suspected renal cell carcinoma as these are the most conservative recommendations [15-16].

If no etiology is elucidated by initial surveillance then intermittent imaging of the patient may be appropriate at the discretion of the physician. If polyarteritis nodosa or another vasculitis is elucidated, further treatment may be necessary based on the specific entity. High-flow vascular malformations may be treated with embolization. If a tumor is found, the extent of the tumor determines the next steps in care. Any renal mass less than 4 cm and restricted to the kidney (T1a) may be managed with biopsy and tumor ablation [16-17]. Treatment of larger tumors or those with metastatic or lymphatic spread may require partial or complete nephrectomy. In our case, No etiology for bleeding was identified despite extensive follow-up [16].

## Conclusions

Spontaneous renal hemorrhage is an uncommon but potentially serious entity defined as active or prior hemorrhage from the kidney in the absence of anticoagulation or inciting trauma. Literature is mixed on appropriate baseline therapy and follow-up guidelines with recommendations ranging from pure clinical management to total nephrectomy. While conservative management should be a major consideration for clinically stable patients, a lower threshold for embolization could have improved outcomes for our patient and should be considered in any case of SRH with evidence of active extravasation. Our suggested algorithm may be a starting point for future research on the subject to clarify the optimal course of treatment.
